# Annual Meeting of the Cook Co. Medical Society

**Published:** 1858-04

**Authors:** 


### ANNUAL MEETING OF THE COOK CO. MEDICAL SOCIETY.

The annual meeting of the Cook County Medical Society was held in the city of Chicago, on Tuesday, April 6th, 1858.

Prof. N. S. Davis, President, in the chair.

The first order of business was the election of officers for the ensuing year, also delegates to the American Medical Association and the Illinois State Medical Society, which resulted as follows:

For President—A. Fisher, M. D.
Vice do.—E. Andrews, M. D.
Secretary—Geo. K. Amerman, M. D.

Delegates to the American Medical Association:

J. W. Freer, M. D. S. Wickersham, M. D.
F. W. White, M. D. C. G. Smith, M. D.
M. O. Heydock, M. D.

The delegates are requested and authorized to appoint substitutes, in case they are unable to attend the meeting at Washington.

Delegates to Illinois State Medical Society were as follows:

E. Powell, M. D. W. H. Byford, M. D.
J. P. Ross, M. D. H. Wardner, M. D.
D. D. Waite, M. D.

The delegates to the Illinois State Medical Society were also empowered and requested to appoint substitutes if necessary. Much other business of a less important character to the profession at large was transacted; after which, the society adjourned.

